# The Sudden Dominance of *bla*_CTX–M_ Harbouring Plasmids in *Shigella* spp. Circulating in Southern Vietnam

**DOI:** 10.1371/journal.pntd.0000702

**Published:** 2010-06-08

**Authors:** Nguyen Thi Khanh Nhu, Ha Vinh, Tran Vu Thieu Nga, Richard Stabler, Pham Thanh Duy, Le Thi Minh Vien, H. Rogier van Doorn, Ana Cerdeño-Tárraga, Nicholas Thomson, James Campbell, Nguyen Van Minh Hoang, Tran Thi Thu Nga, Pham Van Minh, Cao Thu Thuy, Brendan Wren, Jeremy Farrar, Stephen Baker

**Affiliations:** 1 The Hospital for Tropical Diseases, Ho Chi Minh City, Vietnam; 2 The Oxford University Clinical Research Unit, The Hospital for Tropical Diseases, Ho Chi Minh City, Vietnam; 3 The Pathogen Molecular Biology Unit, The London School of Hygiene and Tropical Medicine, London, United Kingdom; 4 Centre for Tropical Medicine, Nuffield Department of Clinical Medicine, Oxford University, Oxford, United Kingdom; 5 EMBL-EBI, Wellcome Trust Genome Campus, Hinxton, Cambridge, United Kingdom; 6 Pathogen Genomics, The Wellcome Trust Sanger Institute, Cambridge, United Kingdom; Massachusetts General Hospital, United States of America

## Abstract

**Background:**

Plasmid mediated antimicrobial resistance in the *Enterobacteriaceae* is a global problem. The rise of CTX-M class extended spectrum beta lactamases (ESBLs) has been well documented in industrialized countries. Vietnam is representative of a typical transitional middle income country where the spectrum of infectious diseases combined with the spread of drug resistance is shifting and bringing new healthcare challenges.

**Methodology:**

We collected hospital admission data from the pediatric population attending the hospital for tropical diseases in Ho Chi Minh City with *Shigella* infections. Organisms were cultured from all enrolled patients and subjected to antimicrobial susceptibility testing. Those that were ESBL positive were subjected to further investigation. These investigations included PCR amplification for common ESBL genes, plasmid investigation, conjugation, microarray hybridization and DNA sequencing of a *bla*_CTX–M_ encoding plasmid.

**Principal Findings:**

We show that two different *bla*_CTX-M_ genes are circulating in this bacterial population in this location. Sequence of one of the ESBL plasmids shows that rather than the gene being integrated into a preexisting MDR plasmid, the *bla*_CTX-M_ gene is located on relatively simple conjugative plasmid. The sequenced plasmid (pEG356) carried the *bla*_CTX-M-24_ gene on an IS*Ecp1* element and demonstrated considerable sequence homology with other *IncFI* plasmids.

**Significance:**

The rapid dissemination, spread of antimicrobial resistance and changing population of *Shigella spp.* concurrent with economic growth are pertinent to many other countries undergoing similar development. Third generation cephalosporins are commonly used empiric antibiotics in Ho Chi Minh City. We recommend that these agents should not be considered for therapy of dysentery in this setting.

## Author Summary

Shigellosis is a disease caused by bacteria belonging to *Shigella spp.* and is a leading cause of bacterial gastrointestinal infections in infants in unindustrialized countries. The *Shigellae* are dynamic and capable of rapid change when placed under selective pressure in a human population. Extended spectrum beta lactamases (ESBLs) are enzymes capable of degrading cephalosporins (a group of antimicrobial agents) and the genes that encode them are common in pathogenic *E. coli* and other related organisms in industrialized countries. In southern Vietnam, we have isolated multiple cephalosporin-resistant *Shigella* that express ESBLs. Furthermore, over two years these strains have replaced strains isolated from patients with shigellosis that cannot express ESBLs. Our work describes the genes responsible for this characteristic and we investigate one of the elements carrying one of these genes. These finding have implications for treatment of shigellosis and support the growing necessity for vaccine development. Our findings also may be pertinent for other countries undergoing a similar economic transition to Vietnam's and the corresponding effect on bacterial populations.

## Introduction

*Enterobacteriaceae* that have the capability to express CTX-M (so named because of their hydrolytic activity against cefotaxime) family extended spectrum beta lactamases (ESBLs) have emerged as a major health threat worldwide [1], [2]. Most of the research in this area is conducted in industrialized countries, where organisms, such as *Escherichia coli* and *Klebsiella spp.*, mostly from urinary tract infections are the commonest source [3], [4], [5]. Relatively little is known about the distribution of such genes in organisms found developing or countries undergoing an economic transition, where the circulating pathogens may differ.

*Enterobacteriaceae* capable of producing ESBLs have been described previously in South East Asia [6], [7]. Ho Chi Minh City in southern Vietnam is typical of many cities where patterns of infectious diseases are changing due to rapid economic growth, better access to health care and improving infrastructure. We recently showed that 42% of healthy people carried ESBL producing bacteria as part of their regular intestinal flora [8]. This previous work suggested that commensal organisms play a role in the dissemination and maintenance of such antimicrobial resistance genes in the population. Furthermore, the uncontrolled use of antimicrobials in the human population and in livestock rearing may lead to further problems with drug resistance and even more limited therapeutic options.

Shigellosis is a gastrointestinal infection caused by members by *Shigella spp.* Due to the faecal oral route of transmission of the *Shigellae*, children less than five years old and living in developing countries have the highest incidence [9], [10]. In our hospital in Ho Chi Minh City, shigellosis is the leading cause of paediatric diarrhoeal admission with bacterial aetiology. The infection is typically self limiting, although antimicrobial treatment is necessary for the young and those that are severely ill as it ensures fewer complications and curtails the duration of the disease [11].

Fluoroquinolones are the drugs of choice to treat *Shigella* infections in both adults and children [12]. However, as with many other members of the *Enterobacteriaceae*, mutations in the genes encoding the target proteins for fluoroquinolones are common in *Shigella* [13], [14]. Our recent findings show that patients with shigellosis are staying in hospital for longer periods compared with 5 and 10 years ago and the disease severity has concurrently increased [15]. Interestingly, at the same time there has been a significant species shift from *S. flexneri* to *S. sonnei* isolated from patients [15]. Patients here are treated with fluoroquinolones, however, those patients that do not respond to the standard therapy are treated with third generation cephalosporins (mainly ceftriaxone). The intravenous third generation cephalosporins are amongst the most commonly used antimicrobials in hospitals in Ho Chi Minh City and the oral second and third generation cephalosporins are also widely available in the community.

Antimicrobial resistance in the *Shigellae* is common; these organisms are closely related to *E. coli* and are readily transformed by exogenous DNA [16], [17], [18]. The distribution of antimicrobial resistance is, however, often different depending on the species. A multi-centre study across Asia demonstrated that *S. flexneri* were more likely to be resistant to ampicillin, whilst *S. sonnei* were more likely to be resistant to co-trimoxazole [19]. Resistance patterns and species dominance are variable depending on the specific location [20], [21], [22].

We have previously reported the rapid emergence of third generation cephalosporin resistant *Shigella* in Vietnam, where we noted the routine isolation of a number of ESBL producing microorganisms [15]. Here, we present data suggesting that ESBL negative organisms have been replaced with ESBL positive organisms.

## Materials and Methods

### Ethics statement

This study was conducted according to the principles expressed in the Declaration of Helsinki. This study was approved by the scientific and ethical committee of the HTD and Oxford tropical research ethics committee (OXTREC) number 010-06 (2006). All parents of the subject children were required to provide written informed consent for the collection of samples and subsequent analysis.

### Patient criteria

The work was conducted on the paediatric gastrointestinal infections ward at the hospital for tropical diseases (HTD) in Ho Chi Minh City in Vietnam. The HTD is a 500 bed tertiary referral hospital treating patients from the surrounding provinces and from the districts within Ho Chi Minh City. All patients from which *Shigella spp.* were isolated were enrolled into a randomized controlled trial comparing treatment with ciprofloxacin and gatifloxicin as described previously [15] (trial number ISRCTN55945881). Briefly, all children (aged 0–14 years) with dysentery (defined as passing bloody diarrhoea or mucoid stools with additional abdominal pain or tenesmus) whose parent or guardian gave fully informed written consent were eligible for admission to the study. The primary outcome of the trial was treatment failure, defined as the patient not clearing symptoms after five days of antimicrobial treatment.

### Microbiological culture and antimicrobial testing

Stool samples were collected from patients and cultured directly on the day of sampling. Samples were cultured overnight in selenite F broth (Oxoid, Basingstoke, UK) and plated onto MacConkey and XLD agar (Oxoid) at 37°C. Colonies suggestive of *Shigella* were sub-cultured on to nutrient agar and were identified using a ‘short set’ of sugar fermentation reactions (Kliger iron agar, urea agar, citrate agar, SIM motility-indole media (Oxoid, United Kingdom)). Serologic identification was performed by slide agglutination with polyvalent somatic (O) antigen grouping sera, followed by testing with available monovalent antisera for specific serotype identification as per the manufacturer's recommendations (Denka Seiken, Japan).

Antimicrobial susceptibility testing of all *Shigella* isolates against ampicillin (AMP), chloramphenicol (CHL), trimethoprim – sulfamethoxazole (SXT), tetracycline (TET), nalidixic acid (NAL), ofloxacin (OFX;) and ceftriaxone (CRO) was performed by disk diffusion (Oxoid, United Kingdom). The minimum inhibitory concentrations (MICs) were additionally calculated for all isolates by E-test, according to manufacturer's recommendations (AB Biodisk, Sweden).

Those strains that were identified as resistant to ceftriaxone using the disk diffusion susceptibility test were further subjected to the combination disc method to confirm ESBL production [23], [24]. The combination disc method utilizes discs containing only cefotaxime (CTX) (30 µg) and ceftazidime (CAZ) (30 µg) and both antimicrobials combined with clavulanic acid (CLA) (10µg). ESBL producing strains were identified as those with a greater than 5 mm increase in zone with the single antimicrobial compared to the combined antimicrobial, i.e. demonstrating ESBL inhibition [25]. All antimicrobial testing was performed on Mueller-Hinton agar, data was interpreted according to the Clinical and Laboratory Standards Institute guidelines [26].

### Genomic DNA isolation and DNA microarray hybridisation

Genomic DNA was isolated from strains that were subjected to PCR and DNA microarray hybridisation from 1 ml of a 5 ml overnight bacterial culture using the wizard genomic DNA extraction kit (Promega, USA), as per the manufacturer's recommendations.

For characterization of gene content of isolated *Shigella* strains, genomic DNA was hybridized to an active surveillance of pathogens (ASP) oligonucleotide microarray [27], [28]. The ASP array contains over 6,000 gene markers, including species signature genes, virulence genes and antimicrobial resistance genes from over a hundred bacterial species. Thus the ASP array provides data for assessing horizontally transferred genes, such data is helpful for diagnosis and for guiding antimicrobial therapy.

The ASP array used in this study was version 6.2 and was designed and constructed as described previously [28]. Test samples were labelled and hybridised as described previously [29]. Briefly, 5 µg genomic DNA was labelled with Cy5 and hybridised with a formamide based hybridisation buffer solution in a final volume of 48 µl at 50°C for 16–20 hours. The ASP arrays were washed as described previously but with the initial wash at 50°C [29]. The ASP arrays were scanned using a 418 microarray Scanner (Affymetrix, USA) and intensity fluorescence data acquired using ImaGene 7.5 (BioDiscovery, USA). Data was analysed as described previously by Stabler *et al.* [28]. Briefly, a reporter was considered positive if the background corrected mean reporter signal from duplicate spots was both greater than one standard deviation of reporter signal (reporter variation) and the mean reporter signal was greater than the whole background corrected microarray mean plus one standard deviation, as shown for *S. sonnei* EG1007 in Dataset S1 in supporting information. The raw microarray data for all isolates is presented in Dataset S2 in supporting information.

### Plasmid extraction and visualisation

Plasmid DNA was isolated from ESBL positive and ESBL negative *Shigella* isolates using a modified version of the methodology previously described by Kado and Liu [30]. The resulting plasmid DNA was separated by electrophoresis in 0.7% agarose gels made with 1× E buffer. Gels were run at 90 V for 3 h, stained with ethidium bromide and photographed. For DNA sequencing plasmid DNA containing an ESBL gene was extracted from an *E. coli* transconjugant using a NucleoBond® Xtra Midi kit as per the manufacturers recommendations (Clontech, USA)

### ESBL gene PCR amplification and characterisation

Genomic DNA was subjected to PCR amplification targeting known classes of *bla* genes using, initially, primers that would recognise sequences encoding SHV, (F; 5′ TCTCCCTGTTAGCCACCCTG, R; 5′; CCACTGCAGCAGCTGC) TEM (F; 5′ TGCGGTATTATCCCGTGTTG, R; 5′ TCGTCGTTTGGTATGGCTTC) and CTX-M (F; 5′ CGATGTGCAGTACCAGTAA, R; 5′ TTAGTGACCAGAATCAGCGG) class ESBLs [31], [32]. Further characterisation of the various sub-group of *bla*_CTX_ ESBL genes was performed using primers, CTX-M-1; (F 5′ ATGGTTAAAAAATCACTGCG, R 5′ TTACAAACCGTCGGTGAC), CTX-M-2; (F 5′ TGGAAGCCCTGGAGAAAAGT and R 5′ CTTATCGCTCTCGCTCTGT) and CTX-M-9; (F 5′ATGGTGACAAAGAGAGTGCAAC, R 5′ TTACAGCCCTTCGGCGATG) using previously outlined PCR amplification conditions [31], [32].

To identify an association with CTX-M genes and the adjacent IS*Ecp1* transposase, all ESBL positive strains were subjected to PCR with primers forward primers Tnp24F 5′ CACTCGTCTGCGCATAAAGCGG, Tnp15F 5′ CCGCCGTTTGCGCATA CAGCGG (for *bla*_CTX-M-24_ and *bla*_CTX-M-15_ respectively) and reverse primer TnpR 5′ AGATATGTAATCATGAAGTTGTCGG. The Tnp24F and Tnp15F were located within the *bla*_CTX-M-24_ and *bla*_CTX-M-15_ genes respectively and TnpR was located within the IS*Ecp1* transposase gene. The *bla*-transposase PCR was performed under the following conditions; 95°C for 1 minute, 30 cycles of 95°C for 30 seconds, 56°C for 30 seconds, 72°C for 1 minute 30 seconds and 72°C for 2 minutes. All PCRs were performed using Taq DNA polymerase and appropriate recommended concentrations of reagents (Bioline, UK).

Positive PCR amplicons were cloned into cloning vector pCR 2.1 (Invitrogen, USA) and sequencing reactions were carried out as recommended by the manufacturer using big dye terminators in forward and reverse orientation on an ABI 3700 sequencing machine (ABI, USA). All sequencing reactions were performed twice to ensure correct sequencing and sequences were verified, aligned and manipulated using Bioedit software (http://www.mbio.ncsu.edu/BioEdit/bioedit.html). All ESBL gene sequences were compared to other ESBL sequences by BLASTn at NCBI. The DNA sequence of various classes of *bla*_CTX_ were downloaded and aligned with the produced sequences.

### Bacterial conjugation

Bacterial conjugation experiments were performed by combining equal volumes (3 ml) of overnight Luria-Bertani cultures of donor and recipient strains. The donor strains were *Shigella* clinical isolates carrying *bla*_CTX_ genes and the recipient was *E. coli* J53 (sodium azide resistant). Bacteria were conjugated for 12 hours at 37°C and transconjugants were selected on Luria-Bertani media containing sodium azide (100 µg/ml) and ceftriaxone (6 µg/ml). Potential transconjugants were verified by serotyping and plasmid extraction.

### Plasmid sequencing and annotation

Plasmid pEG356 was selected for DNA sequencing and annotation as previously described [33]. The DNA sequence was annotated to identify coding sequences and repeat sequences in Artemis. To identify plasmids with similar sequences, pEG356 was compared by BLASTn at NCBI. pAPEC-01-ColBM (Ac. DQ381420) [34] was downloaded and aligned with pEG356 and viewed in Artemis Comparison Tool (ACT) [35]. Schematic drawing of the sequence of pEG356 was constructed using DNAplotter [36]. Artemis, ACT and DNAplotter are freely available at (http://www.sanger.ac.uk/Software). The full sequence and annotation of pEG356 was submitted to EMBL with the accession number FN594520.

## Results

### The escalating isolation rate of ESBL positive *Shigella spp.* in Ho Chi Minh City

During a 24 month period between April 2007 and March 2009 we isolated 94 *Shigella* strains from the stools of children admitted with dysentery. Of these 94 strains, 24 were *S. flexneri* and 70 were *S. sonnei*, confirming the species substitution previously noted from isolates in this region [15]. The general antibiotic sensitivity patterns in these strains were variable, although resistance to trimethoprim – sulfamethoxazole, tetracycline and latterly nalidixic acid were ubiquitous and there was an overall propensity of sensitivity towards older generation antimicrobials such as chloramphenicol (Table 1). A reversion of sensitivity to older therapies highlights how antimicrobial resistance genes can be maintained (or otherwise) by selective antimicrobial pressure in the population.

**Table 1:** Resistance profiles and isolation date of ceftriaxone resistance *Shigella* from southern Vietnam.

								Antimicrobial Tested													
								AMP		CHL		SXT		TET		NAL		OFX		CRO	
Strain ID	Serotype	Age (months)	Sex	Month	Year	Province	ESBL (+/−)	Disc	MIC	Disc	MIC	Disc	MIC	Disc	MIC	Disc	MIC	Disc	MIC	Disc	MIC
DE0611	*S. sonnei*	10	M	February	2001	HCMC	+	R	>256	R	8	R	>32	R	128	S	2	S	0.06	R	>255
EG0356	*S. sonnei*	48	M	May	2007	HCMC	+	R	>256	S	6.0	R	>32	R	64	R	64	S	0.38	R	>256
EG0373	*S. sonnei*	30	M	June	2007	HCMC	+	R	>256	S	6.0	R	>32	R	128	S	1.5	S	0.064	R	>256
EG0384	*S. sonnei*	36	M	July	2007	HCMC	+	R	>256	S	6	R	>32	R	256	R	32	S	0.38	R	>256
EG0390	*S. sonnei*	17	M	August	2007	VINH LONG	+	R	>256	S	6	R	>32	R	128	R	>256	S	0.38	R	>256
EG0395	*S. sonnei*	36	F	September	2007	HCMC	+	R	>256	S	12	R	>32	R	96	R	>256	S	0.5	R	>256
EG0162	*S. sonnei*	28	M	October	2007	DONG THAP	+	R	>256	S	8	R	>32	R	48	R	64	S	0.38	R	>256
EG0419	*S. flexneri*	23	F	December	2007	HCMC	−	R	>256	R	>256	R	>32	R	48	R	>256	S	0.5	R	128
EG0187	*S. sonnei*	16	M	January	2008	DONG THAP	+	R	>256	S	3	R	>32	R	192	S	1.5	S	0.047	R	24
EG0421	*S. sonnei*	36	F	January	2008	HCMC	+	R	>256	S	4	R	>32	R	>256	R	128	S	0.38	R	>32
EG0424	*S. sonnei*	48	F	January	2008	HCMC	+	R	>256	S	6	R	>32	R	64	R	>256	S	0.38	R	>256
EG0204	*S. sonnei*	26	F	March	2008	DONG THAP	+	R	>256	S	6	R	>32	R	32	R	64	S	0.38	R	>256
EG0430	*S. sonnei*	36	F	March	2008	HCMC	+	R	>256	S	6	R	>32	R	>256	R	48	S	0.25	R	128
EG1008	*S. sonnei*	18	M	May	2008	LONG AN	+	R	>256	S	8	R	>32	R	96	R	128	S	0.38	R	>256
EG1009	*S. sonnei*	8	M	May	2008	HCMC	+	R	>256	S	8	R	>32	R	96	R	192	S	0.38	R	>256
EG1010	*S. sonnei*	60	F	May	2008	HCMC	+	R	>256	S	6	R	>32	R	96	R	>256	S	0.5	R	>256
EG1013	*S. sonnei*	25	M	June	2008	HCMC	+	R	>256	S	6	R	>32	R	96	R	>256	S	0.25	R	>256
EG1012	*S. sonnei*	15	F	June	2008	HCMC	+	R	>256	S	8	R	>32	R	96	R	192	S	0.38	R	>256
EG1011	*S. sonnei*	108	F	June	2008	HCMC	+	R	>256	S	8	R	>32	R	96	R	128	S	0.38	R	>256
EG1007	*S. sonnei*	48	M	July	2008	LONG AN	+	R	>256	S	6	R	>32	R	64	R	48	S	0.38	R	192
EG0250	*S. sonnei*	35	M	August	2008	DONG THAP	+	R	>256	S	6	R	>32	R	48	R	48	S	0.25	R	>256
EG0250a	*S. sonnei*	36	M	September	2008	DONG THAP	+	R	>256	S	6	R	>32	R	48	R	48	S	0.25	R	>256
EG0471	*S. flexneri*	49	M	September	2008	HCMC	+	R	>256	R	>256	R	>32	R	128	R	>256	S	0.5	R	>256
EG0472	*S. sonnei*	66	M	September	2008	HCMC	+	R	>256	S	4	R	>32	R	96	R	48	S	0.38	R	>256
EG1014	*S. sonnei*	29	M	January	2009	LONG AN	+	R	>256	S	6	R	>32	R	>256	R	>256	S	0.25	R	>256
EG1015	*S. sonnei*	72	F	January	2009	HCMC	+	R	>256	S	4	R	>32	R	32	R	48	S	0.25	R	>256
EG1016	*S. sonnei*	39	M	January	2009	HCMC	+	R	>256	S	6	S	0.38	S	1.5	R	48	S	0.25	R	>256
EG1017	*S. sonnei*	11	F	February	2009	HCMC	+	R	>256	S	5	R	>33	R	97	R	49	S	1.38	R	>256
EG1018	*S. sonnei*	29	M	February	2009	HCMC	+	R	>256	S	6	R	>32	R	48	R	>256	S	0.38	R	>256
EG1019	*S. sonnei*	120	F	February	2009	HCMC	+	R	>256	S	6	R	>32	R	>256	R	48	S	0.25	R	>256
EG1020	*S. sonnei*	48	M	March	2009	HCMC	+	R	>256	S	8	R	>32	R	64	R	192	S	0.38	R	>256
EG1021	*S. sonnei*	20	M	March	2009	HCMC	+	R	>256	S	8	R	>32	R	64	R	>256	S	0.25	R	>256
EG1022	*S. sonnei*	29	M	March	2009	HCMC	+	R	>256	S	8	R	>32	R	48	R	>256	S	0.25	R	>256
EG1023	*S. sonnei*	9	F	March	2009	LONG AN	+	R	>256	S	6	R	>32	R	48	R	96	S	0.38	R	>256
EG1024	*S. sonnei*	84	M	March	2009	LONG AN	+	R	>256	S	6	R	>32	R	64	R	96	S	0.25	R	>256
EG1025	*S. sonnei*	30	M	March	2009	LONG AN	+	R	>256	S	6	R	>32	R	48	R	96	S	0.25	R	>256

Ampicillin (AMP), chloramphenicol (CHL), trimethoprim – sulfamethoxazole (SXT), tetracycline (TET), nalidixic acid (NAL), ofloxacin (OFX) and ceftriaxone (CRO).

**Figure 1:**
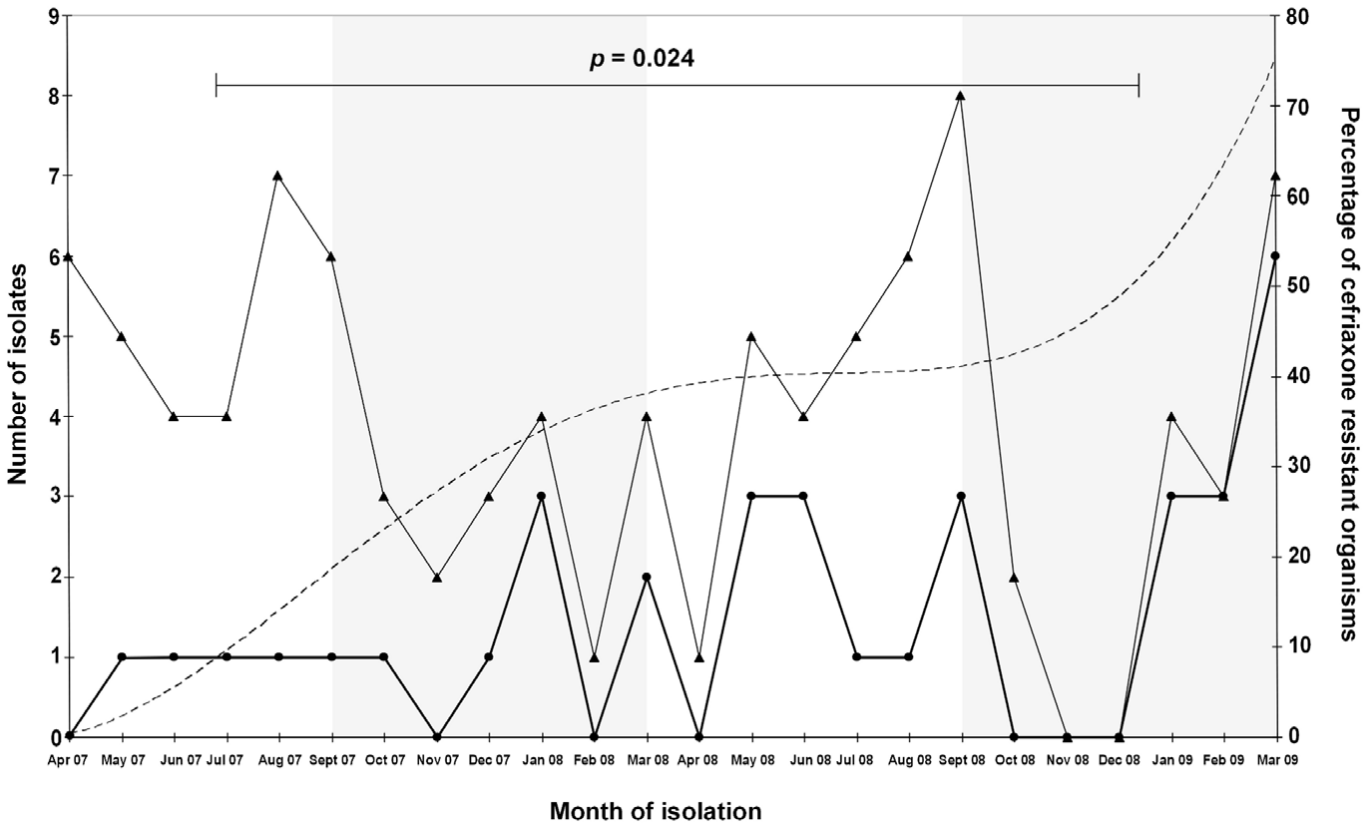
Graph depicting an increase in number and proportion of ceftriaxone resistant *Shigella spp.* isolated between April 2007 and March 2009 at the hospital for tropical diseases in Ho Chi Minh City. The thick black line with circles represents the number of ceftriaxone resistant *Shigella* isolates per month (*x* axis); the thin black line with triangles represents the total number of *Shigella* isolates per month (both related to the left *y* axis). The broken line represents the proportion of strains isolated in six month periods resistant to ceftriaxone (right *y* axis). The increasing proportion of ceftriaxone resistant organisms over six month periods is statistically significant (*p* = 0.024) as calculated using the chi-squared test.

The first isolation of a ceftriaxone resistant organism during the transitional period occurred in May 2007 and similar strains were isolated in low numbers for the following months (Figure 1). The numbers of *Shigellae* isolated that were resistant to ceftriaxone fluctuated over the following 18 months. However, there was increase in the proportion of resistant to sensitive isolates 19% to 41% (5 to 11) between the periods from April 2007–September 2007 and April 2008–September 2008, respectively. This trend peaked in March 2009, with six out of seven *Shigella* strains isolated resistant to ceftriaxone (MIC>256). The overall rate of resistance to ceftriaxone between September 2008 and March 2009 was 75%.

### The combined resistance patterns of ESBL producing *Shigella spp.*

We initially cultured a ceftriaxone resistant *S. sonnei* strain in 2001 (DE 0611) (Table 1), however, this strain was a single, isolated organism and a secondary ceftriaxone resistant *Shigella* was not isolated again until 2007. Between 2007 and 2009, 35 (34%) *Shigella* isolates cultured were resistant to ceftriaxone (Table 1). Of these strains, 33 were *S. sonnei* and the other two isolates were *S. flexneri*. In total, we isolated 36 ceftriaxone resistant organisms between 2001 and 2009.

The mechanism of ceftriaxone resistance was examined by the double disc inhibition method to identify ESBL producing organisms. All the *S. sonnei* and one *S. flexneri* strain (35 from 36 ceftriaxone resistant *Shigella*) produced the characteristic ESBL pattern on investigation, whereas the hydrolysing activity of the other *S. flexneri* organism was not inhibited by clavulanic acid [23], [24] (Table 1).

The median age of patients harbouring third generation cephalosporin resistant *Shigellae* was 32 months (range; 8 to 120 months), the median age of shigellosis patients during the same period was 30 months [15]. Owing to the rapid increase in the rate isolation of such organisms we hypothesised that an individual dominant strain had began circulating in one area of Ho Chi Minh City. However, residence data procured on the time of admission showed that such strains were circulating over a wide area of the city and not purely limited to an isolated outbreak (Table 1). 12 patients were resident in surrounding provinces, some 150 km from the hospital.

In conjunction with ceftriaxone, all strains were examined for resistance to an additional five antimicrobials by disc diffusion and MIC (Table 1). As predicted, all strains demonstrated co-resistance to ampicillin. Thirty five of the 36 strains (97%) were resistant to trimethoprim – sulfamethoxazole and tetracycline, whilst 33/36 were resistant to nalidixic acid. Only three isolates; DE0611, EG0419 and EG0471 were co-resistant to chloramphenicol, of which two, EG0419 and EG0471 (6%), were resistant to five of the six antimicrobials tested (Table 1).

### Identifying the genetic nature of ceftriaxone resistance in *Shigella spp.*

The most common mechanism of dissemination of ESBL genes in the *Enterobacteriaceae* is plasmid mediated transfer. Our previous studies have suggested that Vietnam (and other parts of South East Asia) may be hotspot for the origin and further transmission of antimicrobial resistant organisms [8], [13], [37], [38]. *Enterobacteriaceae* which carry MDR plasmids are common in Vietnam and the isolation of MDR *Shigella* strains has been repeatedly reported [19], [20], [39].

**Figure 2:**
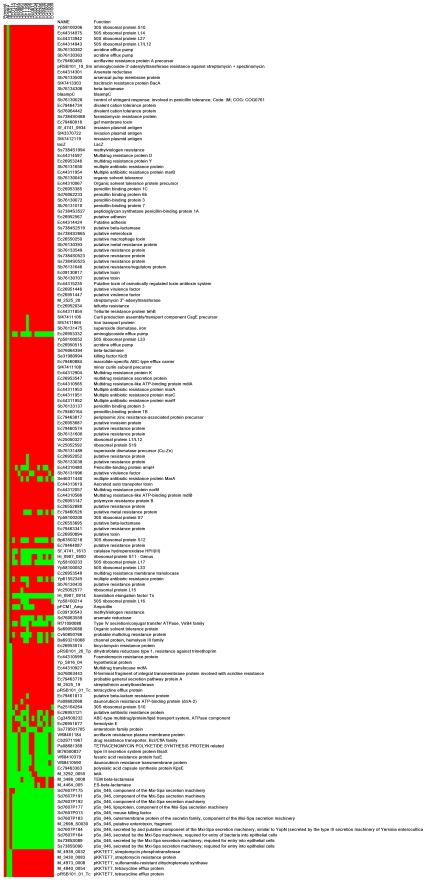
Demonstration of the absence and presence of genes from DNA isolated from ceftriaxone resistant and ceftriaxone sensitive *S. sonnei* isolates using the ASParray. Red boxes indicate presence of genes; green boxes indicate absence of genes. BLAST indicates reporter DNA identity (%) to the *S. sonnei* Ss046 genome. DNA was hybridized from isolates (left to right) DE0115, DE0477, DE0685, DE0891, DE1150, DE1198, DE1256, DE0611, EG0204, EG0373, EG0395, EG0430, EG1007, EG1008 and EG1009.

We hypothesised that the ESBL phenotype was related to the insertion of a transposon carried on an MDR plasmid that had permeated into and was circulating within the *Shigella* population. To investigate the genetic nature of the ESBL positive isolates compared to the ESBL negative isolates we hybridised genomic DNA to an active surveillance of pathogens (ASP) DNA microarray. In total, 15 isolates (seven ESBL positive and eight ESBL negative) were compared. The ASP array is designed to monitor gene flux, genetic content and the nature of horizontally transferred DNA in a bacterial population. The resulting hybridisation is shown in Figure 2. Concurrently, plasmid DNA was isolated and compared from the same bacterial isolates to assess plasmid content.

Figure 2 is a heatmap representation of the 142 ASP microarray reporters which demonstrated positive hybridisation to DNA in two or more of the *S. sonnei* samples and the 11 reporters representing the *S. sonnei* Ss046 plasmid pSS_046. The overall hybridisation data and the names and predicted functions of the genes are presented in Dataset S2 (supporting information).

The pattern of relative hybridisation across all strains was remarkably homogenous, with only 30% (42/142+11 pSS_046) of the total proportion of the positive coding sequences demonstrating variable hybridisation patterns. The coding sequences demonstrating common hybridisation patterns across all 15 strains included a number of signature *E. coli*, *Shigella spp.* regions and sequences corresponding to virulence and antimicrobial resistance (Figure 2 and Supporting information Datasets S1 and S2).

The common antimicrobial resistance genes identified between isolates included genes conferring resistance to streptomycin, macrolides, tetracycline, beta lactams and also some unspecific antimicrobial resistance efflux genes. The homogenous nature of hybridisation suggests that variation between isolates is limited and dependent on plasmid content. All the ESBL producing strains demonstrated significant hybridisation to sequences corresponding to *bla* genes, highlighted in Figure 2, DNA from the ESBL negative strains failed to hybridise to these targets.

Plasmid visualisation of plasmid DNA by agarose gel electrophoresis with all hybridised strains revealed that in contrast to the ESBL negative isolates, all the ESBL producing isolates had a large plasmid, we roughly estimated to be greater than 63 Kbp in size (according to the marker plasmid). Despite the ESBL negative isolates lacking a large plasmid; these strains demonstrated similar resistance profiles, with the obvious exception of ceftriaxone (data not shown). These data suggested that the ESBL genes may be located on simple (none MDR) extrachromosomal elements. This hypothesis was supported by evidence of *in vivo* horizontal plasmid transfer; two strains cultured two days apart from the same patient were identical in serotype, plasmid content and MIC resistance profile, with the exception of the secondary strain carrying a large plasmid and displaying resistance to ceftriaxone (data not shown). Furthermore, sequencing of a conjugative, ESBL encoding plasmid confirmed our suggestion of a simple extrachromosomal element.

### Characterisation of *bla* genes

PCR was performed to detect the *bla*_TEM_, *bla*_SHV_ and *bla*_CTX-M_ genes. Further PCR amplifications were performed on DNA from all strains that produced amplicons with the *bla*_CTX-M_ primers. Primers that were specific for the three major CTX-M clusters, *bla*_CTX-M-9_, *bla*_CTX-M-1_ and *bla*_CTX-M-2_ were selected [40]. Three strains (DE0611, EG0187 and EG0356) produced amplicons with the *bla*_CTX-M-9_ primers and the remaining 32 isolates produced amplicons with the *bla*_CTX-M-1_ primers (Table 2). All 35 PCR amplicon were sequenced.

**Table 2:** Characterisation of *bla*_CTX-M_ genes and the corresponding plasmids of ESBL expressing *Shigella spp.*

Strain ID	Ceftazidime zone (mm)	*bla*_CTX-M_	Plasmid size (kbp)^a^	Conjugation frequency^b^	*bla* -transposon PCR (+/−)
DE0611	28	CTX-M-24	70	4.43×10 ^−2^	+
EG0162	18	CTX-M-15	100	2.73×10 ^−2^	+
EG0187	27	CTX-M-24	70	2.58×10 ^−2^	+
EG0204	19	CTX-M-15	100	1.93×10 ^−2^	+
EG0250	19	CTX-M-15	100	4.43×84 ^−2^	+
EG0250a	19	CTX-M-15	100	4.00×84 ^−2^	+
EG0356	28	CTX-M-24	70	2.41×10 ^−2^	+
EG0373	18	CTX-M-15	100	1.50×10 ^−2^	+
EG0384	20	CTX-M-15	100	2.92×10 ^−2^	+
EG0390	22	CTX-M-15	100	1.38×10 ^−2^	+
EG0395	20	CTX-M-15	100	2.33×10 ^−2^	+
EG0421	20	CTX-M-15	100	1.83×10 ^−4^	+
EG0424	21	CTX-M-15	100	3.77×10 ^−3^	+
EG0430	21	CTX-M-15	100	2.00×10 ^−4^	+
EG0471	20	CTX-M-15	100	1.38×10 ^−2^	+
EG0472	20	CTX-M-15	100	3.59×10 ^−3^	+
EG1007	22	CTX-M-15	100	1.60×10 ^−2^	+
EG1008	20	CTX-M-15	100	1.43×10 ^−2^	+
EG1009	21	CTX-M-15	100	3.11×10 ^−5^	+
EG1010	21	CTX-M-15	100	1.82×10 ^−2^	+
EG1011	21	CTX-M-15	100	5.68×10 ^−6^	+
EG1012	20	CTX-M-15	100	2.37×10 ^−2^	+
EG1013	19	CTX-M-15	100	4.88×10 ^−6^	+
EG1014	19	CTX-M-15	100	2.50×10 ^−3^	+
EG1015	22	CTX-M-15	100	2.75×10 ^−3^	+
EG1016	20	CTX-M-15	100	3.00×10 ^−4^	+
EG1017	20	CTX-M-15	100	3.20×10 ^−2^	+
EG1018	20	CTX-M-15	100	1.45×10 ^−2^	+
EG1019	20	CTX-M-15	100	2.00×10 ^−2^	+
EG1020	20	CTX-M-15	100	0	+
EG1021	21	CTX-M-15	100	1.85×10 ^−3^	+
EG1022	21	CTX-M-15	100	3.75×10 ^−2^	+
EG1023	21	CTX-M-15	100	8.57×10 ^−4^	+
EG1024	20	CTX-M-15	100	3.43×10 ^−2^	+
EG1025	20	CTX-M-15	100	2.36×10 ^−2^	+

a Estimated plasmid size by agarose gel electrophoresis with known markers.

b Conjugation frequency calculated per donor cell from the mean of two replicates.

Sequence analysis of the PCR amplicons demonstrated that there were two differing *bla*_CTX-M_ genes present in the *Shigella* population, these were, *bla*_CTX-M-24_ (n = 3, 8%) and *bla*_CTX-M-15_ (n = 32, 92%) (Table 2). Both genes (*bla*_CTX-M-24_ and *bla*_CTX-M-15_) share 74% DNA homology with each other; *bla*_CTX-M-15_ and *bla*_CTX-M-24_ differ by 12 and 6 nucleotides from the precursor genes within their respective parent groups, (*bla*_CTX-M-1_ and *bla*_CTX-M-9_).

Plasmid sizing, by visualisation of the previous agarose gel electrophoresis demonstrated that the estimated plasmid size corresponded with either the *bla*_CTX-M_ gene (Table 2); *bla*_CTX-M-15_ was consistently located on a plasmid larger than that associated with *bla*_CTX-M-24_. These observations were confirmed by Southern blotting hybridisation of plasmid DNA extractions (data not shown). The differing plasmid sizes and ESBL genes correlated precisely with two distinct zone clearance areas when strains were susceptibility tested with ceftazidime. The strains expressing CTX-M-24 demonstrated less activity against ceftazidime when compared to CTX-M-15 (median zone size, CTX-M-24; 28mm, CTX-M-15; 20mm) (Table 2).

All *bla*_CTX-M_ harbouring plasmids with the exception of the plasmid in strain EG1020 were transmissible with high conjugation frequencies, ranging from 4.84×10^2^ to 4.88×10^6^ (median 1.55×10^2^) per donor cell (Table 2). The mobilisation of one of these *bla*_CTX_ harbouring plasmids was further demonstrated by conjugative transfer of the plasmid originally from *S. sonnei* EG356 from an *E.coli* transconjugant back into a fully susceptible, naive *S. sonnei* strain at a similarly high frequency.

### DNA sequence analysis of the pEG356 plasmid

The ESBL encoding gene *bla*_CTX-M-24_ appears to be generally restricted to *Enterobacteriaceae* in Asia [41], [42], with only sporadic reports of this gene in other locations [43]. Therefore, we selected the plasmid from isolate EG0356, carrying a *bla*_CTX-M-24_, as it is applicable to this location, for further characterisation by DNA sequencing.

**Figure 3:**
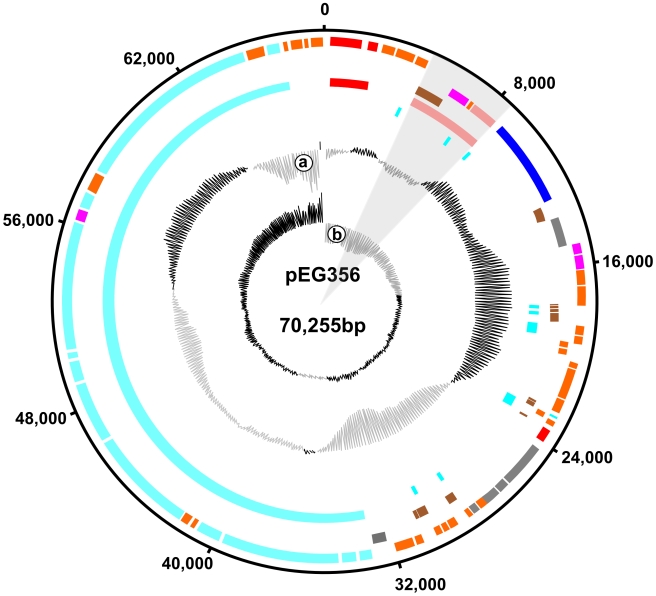
A schematic representation of the *bla*_CTX-M-24_ encoding plasmid, pEG356. pEG356 is a 70,275bp *IncFI* plasmid containing 104 coding sequences. The various features are highlighted by the various concentric circles according to the annotation of the of the plasmid (ac. FN594520). The outer colored circle represents coding sequences on the forward strand, the second circle represents coding sequences on the reverse strand. The coding sequences are coded by colour, red; plasmid replication, orange; conserved hypothetical, brown; pseudogene, dark blue; adaptation, grey; segregation, light blue; conjugation/transfer, light pink; transposition, dark pink; degradation/resistance and yellow; metabolism. The third concentric circle represents the location of pseudogenes and the fourth circle represents the four main modules of predicted function, red; replication, pink; transposition, dark blue; iron transport and light blue; conjugational transfer. The fifth and final coloured circle represents the location of the repeat sequences. The primary central graph (a) represents GC content, ranging from high (black) to low (grey) (mean 52%) and the secondary central graph (b) represents G/C coding bias ranging from high (black) to low (grey). The IS*Ecp1* type element carrying the *bla*_CTX-M-24_ is distinguished by grey shading.

Plasmid pEG356 was found to be a circular replicon consisting of 70,275 nucleotides, similar in size to another *bla*_CTX-M-24_ encoding plasmid from Asia; pKP96. pKP96 was isolated from a *Klebsiella pneumoniae* strain from China in 2002, yet demonstrates limited DNA homology to pEG356, with exception to the ESBL encoding region [44]. pEG356 was comparatively GC neutral (52.26%) and belonged to incompatibility group *FI* (on the basis of the DNA sequence homology to the replication region) (Figure 3). pEG356 was predicted to contain 104 coding sequences, of which 14 were considered to be pseudogenes on the basis of apparent premature stop codons, frameshifts or missing start codons. The density of coding sequencing approached 95% and contained four main structural features, a replication region, the ESBL gene encoding region with predicted homology to an IS*Ecp1* element, an iron ABC transport system and a DNA transfer region (labelled red, pink, dark blue and light blue, respectively in Figure 3).

pEG356 encoded the complete *tra* gene-set encoding a conjugative pilus with high sequence similarity to the transfer region from the F plasmid sequence from *E. coli* K12 [45] (Ac. AP001918). This is consistent with the *in vitro* data demonstrating that this plasmid is transmissible into an *E. coli* recipient. The *IncFI* replication region was highly similar to other *IncF* plasmids, including the recently described CTX-M-15 encoding plasmid pEK499 (Ac. EU935739) isolated from an *E. coli* O25:H4-ST131 epidemic strain circulating in the United Kingdom [46]. Additionally, pEG356 shared another 30 Kbp (position 15,152 to 44,255 in pEG356) of high sequence similarity with pEK499 [46]. This region contains multiple common hypothetical plasmid genes of unknown function, genes involved in conjugative transfer (*traM* to *traC*), plasmid partitioning and a predicted single stranded DNA binding protein (*ssb*). Unlike pEK499 the *mok* and *hok* post segregational killing genes are missing from within the plasmid maintenance region [46]. With respect to pEK499 and other ESBL carrying plasmids, pEG356 does not carry multiple antimicrobial resistance genes, transposons, insertion sequences or any additional virulence associated genes [44], [46], [47](Chen et al. 2007; Shen et al. 2008; Woodford et al. 2009)(Chen et al. 2007; Shen et al. 2008; Woodford et al. 2009).

**Figure 4:**
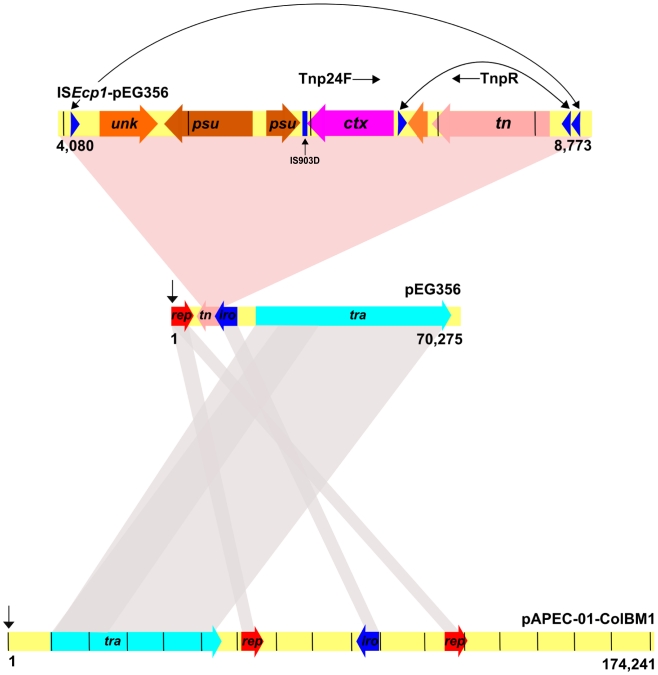
A schematic representation of IS*Ecp1*-pEG356 and DNA sequence alignment highlighting corresponding DNA homology between pEG356 and pAPEC-O1-ColBM. The DNA sequences for pEG356 and pAPEC-O1-ColBM were aligned and compared in Artemis comparison tool (ACT). The numbers on the diagram correspond to the respective plasmid sizes and the black integers highlight 10 Kbp intervals. The genetic backbone of the pEG356 and pAPEC-O1-ColBM is shown in yellow along with the various modules, red; *rep* (replication), pink; *tn* (transposition), dark blue; *iro* (iron uptake) and light blue; *tra* (DNA transfer/conjugation). Areas with high DNA homology between pEG356 and pAPEC-O1-ColBM are shown with grey shading and the pink shading corresponds to a magnified view of IS*Ecp1*-pEG356. The numbers on IS*Ecp1*-pEG356 correspond to the location of the element on the host plasmid, with integers representing 1 Kb intervals. The genes are functionally coded, pink; *tn* (transposition), orange; *unk* (unknown function), brown; *psu* (pseudogene) and dark pink; *bla*_CTX-M-24_, primer locations for the transposon PCR are highlight by Tnp24F and TnpR. The location of the IS908D downstream of the *bla*_CTX-M-24_ is highlighted The region is flanked by an inverted repeat (blue triangles) and contains an additional inverted repeat sequence flanking the transposase gene. Corresponding inverted repeats are linked by arrows.

In overall structure, but not size, pEG356 shared the most DNA sequence similarity with the ColBM plasmid pAPEC-O1 (Ac. DQ381420), isolated from an avian pathogenic *E. coli* strain [34] (Figure 4). pEG356 shared around 80% of the gene content with pAPEC-O1, including the conjugation (*tra*), replication (*rep*) and a putative ATP iron transport system (*iro*). The *iro* region consisted of four coding sequences, which include, a putative permease, an iron binding protein and an export associated protein.

The *bla*_CTX-M-24_ was located on an IS*Ecp1* like element. The overall sequence of the IS*Ecp1* variant on pEG356 is 4,725 bp and 3,000 bp shares 99% DNA homology with an ESBL gene encoding element from an *E. coli* strain that was isolated in China; pOZ174 (AF252622) [48]. The *bla*_CTX-M-24_ carrying region is also highly similar (99% DNA homology) to the equivalent region in the previously described plasmid, pKP96, including the IS903D downstream of the *bla*_CTX-M-24_ gene (Figure 4) [44].The IS*Ecp1* element contains two pairs of inverted repeat (Figure 4): the larger inverted repeat (31 bp) flanks the complete element, inclusive of six coding sequences. The 3′end of the IS*Ecp1* element contained a IS*Ecp1* transposase and a small hypothetical coding sequence of unknown function which is spanned by two IS1380 elements. The *bla*_CTX-M-24_ isadjacent to two pseudogenes, which were understood to have encoded a conserved hypothetical transposon protein and a maltose-inducible porin precursor, it is not clear what significance, if any, these genes are to the overall functionality of the element or the plasmid.

All ESBL producing *Shigella* were subjected to PCR to demonstrate if all *bla* genes were associated with the IS*Ecp1* transposase. The location of the PCR primers Tnp24F and TnpR are highlighted in Figure 4 and were designed to produce an amplicon if the *bla* gene and the adjacent IS*Ecp1* transposase were in the same location and orientation in strains with a *bla*_CTX-M-24_. A secondary forward primer was designed in equivalent location for those strains with a *bla*_CTX-M-15_ (Tnp15F). Therefore, if *bla*_CTX-M-24_ or the *bla*_CTX-M-15_ was consistently adjacent to the IS*Ecp1* transposase it would produce an amplicon of 414 bp in all strains. All ESBL positive strains (CTX-M-15 and CTX-M-24) generated a PCR amplicon of the predicted size (Table 2). Sequencing of all PCR products demonstrated that all the *bla*_CTX-M-15_ and the *bla*_CTX-M-24_ gene were associated with an IS*Ecp1* transposase, The DNA sequence from all PCR products was identical from within the transposase gene up to and including the IS1380.

## Discussion

Members of the *Enterobacteriaceae* that carry CTX-M family ESBLs have been isolated from many parts of the world since the mid 1990s [40]. CTX-M genes have been previously identified from pathogenic *Enterobacteriaceae* circulating in South East Asia; such as Vietnam, Thailand, Cambodia and Singapore [6], [7], [49], [50]. Additionally, our work has shown that ESBLs are commonly found in organisms which constitute the “normal” gastrointestinal flora in the general population living in Ho Chi Minh City [8]. Such data predicts that intestinal flora may be a considerable reservoir of ESBL encoding genes and the genetic elements they circulate on, permitting potential transmission to their pathogenic counterparts.

CTX-M genes in the *Shigellae* have been previously reported in Argentina, (CTX-M-2) [51], Korea (CTX-M-14) [52] and from a traveler returning from India (CTX-M-15) [53]. More recently, Nagano *et al.* described a novel CTX-M-64 hybrid from a shigellosis patient infected with *S. sonnei* after returning to Japan from China [54]. The *S. sonnei* strains isolated here in Ho Chi Minh City harbored the *bla*_CTX-M-15_ and *bla*_CTX-M-24_ genes. Current data suggests that *bla*_CTX-M-24_ is found mainly in Asia [41], [42], yet may have been transferred to other locations [43]. MDR CTX-M-15 producing *E. coli* is emerging worldwide as an important pathogen causing hospital-acquired infections [2]. The potential impact of MDR *Shigella* combined with CTX-M-15/24 carrying plasmids is substantial, with implications for local treatment policy and the transportation of such plasmids into other countries as has been implicated in Canada [43], [55].

The structure of pEG356 as a vector for transferring *bla*_CTX-M-24_ implies that such plasmids may be common. The streamlined nature of pEG356, remarkably high conjugation frequency may ensure onward circulation of the genetic cargo as it becomes stable in the bacterial population. The simplistic nature of pEG356, with a lack of additional resistance genes suggests that this is a contemporary element, with the *bla*_CTX-M-24_ a recent acquisition. The *bla*_CTX-M-24_ gene has been located on a relatively uncomplicated plasmid in Asia, however, pKP96 only demonstrates limited homology to pEG356 [44].

All ESBL gene were located adjacent to a IS*Ecp1* transposase (as identified by PCR). We are currently unable to substantiate if it is the IS*Ecp1*-like element, the plasmids or the circulation of bacterial clone is responsible for the increasing rate of isolation. However, the geographical spread of these strains suggests that they are widely disseminated throughout southern Vietnam. *S. sonnei* is a monophyletic bacterial pathogen, and owing to the lack of sensitivity of existing sequence based methods such as multi locus sequence typing [56], we are currently unable to confirm clonality satisfactorily (data not shown). Further epidemiological investigation of CTX-M containing strains combined with a more sensitive sequenced based methodology, such as is used for *Salmonella* Typhi is required [57]. We are currently assessing the genetic nature of the strain and the plasmids carrying the ESBL genes.

Our findings show a transfer from 0% to 75% ceftriaxone resistance in *S. sonnei* over just two years in the key age group (1 to 3 years) for this disease. By sampling across the Ho Chi Minh City area, covering approximately 150 sq kilometres of Vietnam and a population of approximately 15 million people we have shown that the genetic explanation for this resistance pattern is the dissemination two distinct ESBL genes, of which one is dominant. These are the leading source of ESBLs in clinical *Shigella* cases and their rapid spread suggests that these organisms are under strong selection pressure. The use of third generation cephalosporins, such as oral cefpodoxime and cefixime in the community is common in Vietnam, and places the even the short term usage of ceftriaxone and other broad-spectrum cephalosporins in jeopardy.

*Shigella spp.* are capable of carrying multiple plasmids with an array of phenotypes including virulence and antimicrobial resistance [16], [18]. The presence of *Shigella* in the gastrointestinal tract of humans is an ideal environment to acquire horizontally transferred genetic material. Small highly transmissible plasmids that impinge on the fitness of the host may be rapidly disseminated under appropriate conditions.

Vietnam is a country that in many respects is representative of many parts of the world. The Vietnamese economy is developing rapidly and the country is undergoing transition with an increasing population, urbanisation and shifting patterns of infectious diseases. In the past decade there has been a transition in species from *S. flexneri* to *S. sonnei* in the Southern provinces of Vietnam. With this shift has come the emergence of ESBL *S. sonnei*. These findings from the Vietnamese population should perhaps serve as a warning for other countries encountering the same economic transition. The progressive evolution of pan-resistant *Shigella* makes vaccine development an increasingly important objective.

## Supporting Information

Alternative Language Abstract S1 Translation of abstract into Vietnamese by Tran Vu Thieu Nga.
(0.04 MB DOC)

Dataset S1 Corrected microarray data mean plus one standard deviation for *S. sonnei* EG1007.
(1.03 MB XLS)

Dataset S2 Raw microarray data for all *S. sonnei* isolates.
(1.03 MB XLS)
