# Supplementary material for: The Sudden Dominance of bla CTX–M Harbouring Plasmids in Shigella spp. Circulating in Southern Vietnam
Source: PLoS Negl Trop Dis. 2010 Jun 8;4(6):e702. doi: 10.1371/journal.pntd.0000702 (PMC2882334; doi:10.1371/journal.pntd.0000702)
Supplement: Alternative Language Abstract S1 — Translation of abstract into Vietnamese by Tran Vu Thieu Nga. (0.04 MB DOC) [file pntd.0000702.s001.doc]

**Cơ sở khoa học**

Kháng kháng sinh qua trung gian plasmid ở họ vi khuẩn đường ruột *Enterobacteriaceae* là một vấn nạn toàn cầu. Việc tiết men beta lactamases phổ rộng (ESBL) họ CTX-M của các vi khuẩn này gia tăng đã được ghi nhận tại các nước phát triển. Việt Nam là một đại diện tiêu biểu cho những quốc gia có thu nhập trung bình mà tại đây hệ thống chăm sóc sức khỏe cộng đồng đang phải đương đầu với những thách thức phát sinh từ sự lan rộng tình trạng kháng thuốc đối với các bệnh truyền nhiễm.

**Phương pháp nghiên cứu**

Chúng tôi thu thập bệnh án của các bệnh nhi nhiễm *Shigella* nhập viện vào Bệnh Viện Bệnh Nhiệt Đới Thành Phố Hồ Chí Minh. Các chủng vi khuẩn phân lập từ tất cả những bệnh nhân tham gia nghiên cứu được kiểm tra tính kháng kháng sinh. Những chủng tiết ESBL được sử dụng làm vật liệu cho những thí nghiệm tiếp theo bao gồm PCR khuyếch đại các gene thường gặp mã hóa cho việc tiết ESBL, điện di plasmid, thí nghiệm tiếp hợp, lai DNA vi bản và giải trình tự plasmid mang gene mã hóa cho *bla*_CTX–M_.

**Những kết quả quan trọng**

Chúng tôi chứng minh sự hiện diện của hai gene *bla*_CTX-M_ khác nhau tồn tại trong quần thể vi khuẩn nghiên cứu. Gene *bla*_CTX-M_  này định vị trên một plasmid tương đối đơn giản thay vì liên kết với một plasmid đa kháng thuốc. Plasmid pEG356 có mang gen *bla*_CTX-M_ trên vùng tương tự nhân tố IS*Ecp1* và cho thấy có sự tương đồng cao với plasmid *IncFI*.

**Kiến nghị**

Tình trạng lan rộng nhanh chóng tính kháng kháng sinh và việc chuyển vị của chủng ưu thế trong quần thể *Shigella* đi cùng với sự phát triển kinh tế là thực trạng chung cho nhiều quốc gia đang phát triển khác. Cephalosporins thế hệ thứ ba là loại kháng sinh được sử dụng rộng rãi không kiểm soát tại Thành Phố Hồ Chí Minh. Nhưng dựa theo kết quả thu được từ nghiên cứu, chúng tôi kiến nghị không dùng kháng sinh này để chữa trị cho bệnh nhân đi tiêu ra máu tại miền Nam Việt Nam.
